# Probability of Spirochete *Borrelia miyamotoi* Transmission from Ticks to Humans

**DOI:** 10.3201/eid2112.151097

**Published:** 2015-12

**Authors:** Denis S. Sarksyan, Alexander E. Platonov, Lyudmila S. Karan, German A. Shipulin, Hein Sprong, Joppe W.R. Hovius

**Affiliations:** Izhevsk State Medical Academy, Izhevsk, Russia (D.S. Sarksyan);; Central Research Institute of Epidemiology, Moscow, Russia (A.E. Platonov, L.S. Karan, G.A. Shipulin);; National Institute of Public Health and the Environment, Bilthoven, the Netherlands (H. Sprong);; Academic Medical Center, Amsterdam, the Netherlands (J.W.R. Hovius)

**Keywords:** Borrelia infections, disease transmission, infectious, Ixodes, bites and stings, Russia, vector-borne infections, ticks, Borrelia miyamotoi

**To the Editor:** Borreliosis caused by *Borrelia miyamotoi* is an emerging disease transmitted by *Ixodes* ticks (*1*). Each year in the Netherlands during 2007–2009, ≈70,000 bites by hard ticks occurred per 1 million inhabitants (*2*). In the Republic of Udmurtia, Russia, ≈10,000 hard tick bites per 1 million inhabitants are reported annually among persons seeking medical help. Recent studies indicate that almost 3% of *I. ricinus* ticks in the Netherlands and 2%–6% of *I. persulcatus* ticks in Russia are infected with *B. miyamotoi* (*1*,*3*,*4*). Human exposure is substantial, and comparable exposure to *B. miyamotoi* is expected in many Eurasian countries and in North America (*4*,*5*). The probability of *B. miyamotoi* transmission from ticks to humans requires examination to estimate the true risk for human disease.

In Izhevsk (population 650,000), a city in European Russia (Republic of Udmurtia), we identified 95 human cases of *B. miyamotoi* infection during 2010–2014 (*6*). In this city, primarily because of concern about tickborne encephalitis (TBE), all patients with suspected tickborne infection are hospitalized in the Republican Hospital of Infectious Diseases (RHID). A service also enables tick-bitten persons to bring the removed tick for PCR for TBE virus (TBEV) and *B. burgdorferi* sensu lato. We supplemented that with PCR testing for *B. miyamotoi* (*3*).

In June 2014, twenty-four persons (≈5% of those bitten by ticks subjected to PCR-based investigation for TBEV, *B. burgdorferi* sensu lato, and *B. miyamotoi*) were bitten by adult *I. persulcatus* ticks infected with *B. miyamotoi* only. We informed these persons of their results and about the clinical features of *B. miyamotoi* infection and recommended self-observation during 2 months (twice the maximum incubation period for *B. miyamotoi* infection [*3*,*6*]). These persons were advised to contact their medical supervisor at RHID (D.S. Sarksyan) if fever, fatigue, erythema migrans, or any other possible symptom of a tickborne infection developed. In 2 patients, such symptoms developed: one 12 days (patient 1), the other 16 days (patient 2), after the tick bite. *B. miyamotoi* DNA was detected by PCR in their blood on admission to RHID. Neither IgM nor IgG were found by a nonspecific ELISA (Omnix, St. Petersburg, Russia [*7*]) that reacts with serum from *B. burgdorferi* sensu lato–infected and *B. miyamotoi*–infected persons. However, *Borrelia* IgM and IgG were detected in serum obtained 12 and 45 days after illness onset from patient 1 and 6 and 39 days later from patient 2. Clinical characteristics were typical of *B. miyamotoi* infection: chills, sweating, headache, dizziness, fatigue, thirst, nausea, vomiting, fever (axillar temperature 38.8°C in patient 1 and 39.0°C in patient 2). Erythema migrans was absent. Both patients had modest thrombocytopenia (134 [patient 1] and 118 [patient 2] × 10^9^ platelets/mL [reference range 150–400 × 10^9^ platelets/mL]) and increased band neutrophils (10% [patient 1] and 8% [patient 2] of leukocytes [reference range 1%–5%]). Patient 2 had clinical and laboratory signs of kidney dysfunction (oliguria, leukocytes, erythrocytes, and epithelial cells in a urine sample), a complication observed in 18% of 95 patients with *B. miyamotoi* disease (*4*). Both patients were treated with doxycycline (100 mg 2×/d) for 10 days; they clinically recovered, and laboratory abnormalities returned to reference ranges at discharge 12 days after admission. 

The remaining 22 persons did not report any malaise and were examined 1 month after tick bite. They appeared healthy at that time, and PCR and ELISA gave negative results, arguing against possible asymptomatic *B. miyamotoi* infection.

We estimated the probability of *B. miyamotoi* transmission to humans to be 8.3% (95% CI 4%–18% using a Bayesian approach [*8*] or 95% CI 0%–21% using an SPSS bootstrapping procedure [SPSS Inc., Chicago, IL, USA]). For comparison, among 68 persons bitten by *B. burgdorferi* sensu lato–infected ticks in the Netherlands, erythema migrans developed in 4.4% (95% C.I. 2.1%–8.3%) persons; 3 (4.4%) others seroconverted without clinical symptoms (*9*).

This pilot study has several limitations. We did not follow up persons bitten by *B. burgdorferi* sensu lato– or TBEV-infected ticks because they received either antimicrobial drugs or anti-TBE immunoglobulin as a preventive measure. Because of labor constrains, we did not study persons bitten by “PCR-uninfected” ticks; however, they were not hospitalized at RHID, the only hospital in the region for patients with evident tick-borne diseases. We did not use any serologic techniques specific for relapsing fever *Borreli* (e.g., GlpQ ELISA). Although we did not test for *Rickettsia* or *Babesia* spp., we did not find TBEV RNA, *B. burgdorferi* sensu lato 16S RNA, pathogenic *Ehrlichia* 16S RNA, or pathogenic *Anaplasma* DNA in the 2 *B. miyamotoi*–positive patients’ blood samples. 

We demonstrated that the transmission rate of *B. miyamotoi* appears to be equal to, or higher, than that of *B. burgdorferi* sensu lato (*1*,*9*,*10*). Our data indicate that, annually, clinical *B. miyamotoi* infection might develop in at least 0.005% of persons living in regions to which *Ixodes* spp. ticks and *B. miyamotoi* are endemic. This estimate corresponds to ≈33 cases annually in Izhevsk, which is similar to the previously published results of hospital-based surveillance for *B. miyamotoi* (*3*,*6*).

## References

[1] Wagemakers A, Staarink PJ, Sprong H, Hovius JW. *Borrelia miyamotoi*: a widespread tick-borne relapsing fever spirochete. Trends Parasitol. 2015;31:260–9. 10.1016/j.pt.2015.03.008 25892254

[2] Hofhuis A, Harms M, van den Wijngaard C, Sprong H, van Pelt W. Continuing increase of tick bites and Lyme disease between 1994 and 2009. Ticks Tick Borne Dis. 2015;6:69–74. 10.1016/j.ttbdis.2014.09.006 25448421

[3] Platonov AE, Karan LS, Kolyasnikova NM, Makhneva NA, Toporkova MG, Maleev VV, Humans infected with relapsing fever spirochete *Borrelia miyamotoi*, Russia. Emerg Infect Dis. 2011;17:1816–23. 10.3201/eid1710.101474 22000350 PMC3310649

[4] Krause PJ, Fish D, Narasimhan S, Barbour AG. *Borrelia miyamotoi* infection in nature and in humans. Clin Microbiol Infect. 2015;21:631–9. 10.1016/j.cmi.2015.02.006 25700888 PMC4470780

[5] Molloy PJ, Telford SR III, Chowdri HR, Lepore TJ, Gugliotta JL, Weeks KE, *Borrelia miyamotoi* disease in the northeastern United States: a case series. Ann Intern Med. 2015;163:91–8. 10.7326/M15-0333 26053877

[6] Sarksyan DS, Platonov AE, Karan LS, Malinin IE, Khalitova LI, Shakhov VI, Clinical presentation of “new” tick-borne borreliosis caused by *Borrelia miyamotoi* [in Russian]. Ter Arkh. 2012;84:34–41 . 23252245

[7] Immunoassay for differential detection of class M and G antibodies to the agents of *Ixodes* tick-borne borreliosis (Lyme disease) [in Russian] [cited 2015 Sep 7]. http://www.omnix.ru/materials/ooKKKWkM.pdf

[8] Estimated true prevalence using one test with a Gibbs sampler [cited 2015 Sep 7]. http://epitools.ausvet.com.au/content.php?page=OneTest

[9] Hofhuis A, Herremans T, Notermans DW, Sprong H, Fonville M, van der Giessen JW, A prospective study among patients presenting at the general practitioner with a tick bite or erythema migrans in the Netherlands. PLoS ONE. 2013;8:e64361. 10.1371/journal.pone.0064361 23696884 PMC3655959

[10] Huegli D, Moret J, Rais O, Moosmann Y, Erard P, Malinverni R, Prospective study on the incidence of infection by *Borrelia burgdorferi* sensu lato after a tick bite in a highly endemic area of Switzerland. Ticks Tick Borne Dis. 2011;2:129–36. 10.1016/j.ttbdis.2011.05.002 21890065

